# A study on the effect of fibromyalgia severity on sleep quality using inflammatory markers

**DOI:** 10.6026/9732063002001608

**Published:** 2024-11-30

**Authors:** Ruchi Singh, Jyotsana Rai, Akash Pathak, Nirendra kumar Rai

**Affiliations:** 1 Department of Physiology, AIIMS Bhopal, Madhya Pradesh, India; 2 Department of Neurology, AIIMS Bhopal, Madhya Pradesh, India

**Keywords:** Fibromyalgia severity and pain intensity, fibromyalgia and C-reactive protein, sleep quality and fibromyalgia syndrome, fibromyalgia severity and inflammatory markers, sleep disturbances and inflammatory markers

## Abstract

Fibromyalgia syndrome (FMS) is a widespread musculoskeletal pain, often accompanied by fatigue and sleep disturbances. Poor sleep exacerbates inflammation and pain, potentially increasing levels of inflammatory markers. This study explored the effect of FMS severity on CRP levels, sleep quality and pain intensity. 89 FMS individuals were explored for FMS severity, pain and sleep quality. CRP levels in blood and erythrocyte sedimentation rate (ESR) were measured to assess inflammation. An increase in FMS severity was associated with an increase in CRP and ESR levels, pain intensity and sleep disturbances. However, a positive correlation between CRP levels and sleep quality indicates that Poor sleep quality in FMS may contribute to elevated CRP levels. Addressing sleep quality may mitigate the severity of FMS and its associated symptoms.

## Background:

Fibromyalgia Syndrome (FMS) is characterized by widespread musculoskeletal discomfort, often accompanied by various other health challenges like fatigue, disrupted sleep patterns and cognitive issues [1]. It's prevalent among middle-aged women, affecting approximately 0.5-5% of the global population [2]. Individuals with FMS are known to present with sleep disturbances as well as poor sleep quality [3, 4]. Inadequate sleep can contribute to increased inflammation and perception to pain within the interrelated triad of sleep, pain and inflammation [5, 6]. Sleep deprivation leads to an increase in the level of inflammatory marker TNF-R p55, a cytokine factor that controls the availability of TNF-α, which has the potential to induce sleep [7]. Notably, acute sleep deprivation and day-to-day reductions in sleep of 25-50% have shown increased levels of interleukin-6 (IL-6) and C-reactive protein (CRP) [8, 9-10]. Individuals with primary insomnia have been shown to exhibit elevated IL-6 levels and PGE2 [11]. Additionally, increased levels of brain PGD synthase, responsible for synthesizing PGD2, have been observed to be associated with increased daytime sleepiness in patients with narcolepsy [12]. Inflammation is frequently modulated by classical prostaglandins like PGE2, but subsequent research has shown the involvement of additional facilitators (TNF-α) and inducers of pain (IL-6) [13]. Haack *et al.* have demonstrated that restriction of sleep to 50% for 12 days leads to higher subjective pain ratings [14]. Thus, the quantity of sleep affects both PG as well as pro-inflammatory cytokines, which play an integral role in the experience of pain [15]. The pathophysiology of pain suggested for FMS primarily highlights alternation in central pain pathways and/or central sensitization (CS), however, studies have also postulated the role of inflammatory processes in FMS pathogenesis [16]. Several studies explored the relationship of CRP in individuals with FMS including Haheim *et al.* who included only male patients of chronic diseases with 137 individuals of FMS, making it difficult to determine the precise nature of association between CRP and FMS [17]. A large cross-sectional study by Feinberg *et al.* investigated the impact of comorbid disorders and BMI on the CRP-FMS association in the US population [18]. Zetterman *et al.* [19] reported significantly higher levels while Groven *et al.* [20]. Observed marginally elevated levels of high-sensitivity C-reactive protein in FMS individuals compared to the control group. Other studies of Xiao *et al.* Bazzichi *et al.* Rus *et al.* though have reported a positive association of CRP with FMS, none of them have mentioned any association of CRP level with the severity of FMS [21, 22-23]. Therefore, it is of interest to describe the association of the severity of FMS with the level of CRP, sleep quality and Pain among individuals with FMS.

## Materials and Methods:

This cross-sectional study was done at a tertiary health care centre. Approval for the study was obtained from the institutional ethical committee and the study was conducted in accordance with the ethical guidelines of the Declaration of Helsinki. Participants were enrolled in the study after a written informed consent and detailed information about the study was provided to them. All questionnaires were comprehensively explained to all the participants who consented for the study.

## Participants:

FMS patients > 18 years of age who met the diagnostic criteria for FMS outlined by the American College of Rheumatology (ACR) in 2010 were enrolled in the study [24]. Pregnant women and individuals with other chronic neurological/psychiatric diseases were excluded from the study.

## Procedures:

After the initial evaluation and examination, demographic characteristics (age, gender, weight, height) were recorded and the participants were asked to fill certain questionnaires which included Fibromyalgia Impact Questionnaire Revised (FIQR) - to evaluate severity of disease, Visual analog scale (VAS) and Global Pain scale(GPS) for Pain and Pittsburgh Sleep Quality Index (PSQI) for sleep. In addition to routine blood examination to rule out any other co-morbidity, Erythrocyte sedimentation rate (ESR) and Inflammatory markers such as CRP were also conducted (Table 1).

## Fibromyalgia impact questionnaire-revised (FIQR):

FIQR was employed to evaluate severity of FMS. It comprised of three domains: function (nine questions), overall impact (two questions) and symptoms (ten questions). All 21 items are rated on an 11-point Likert scale from 0 to 10. Total score of function domain was divided by 3 and the total score of symptoms domain was divided by 2. The final FIQR score is the sum of these three domain values, with a maximum score of 100 with higher scores indicating more severe symptoms [25]. The severity of fibromyalgia is categorized as remission (≤30), mild severity (>30 and ≤45), moderate severity (>46 and ≤65) and high severity (>65).

## Goble pain scale and visual analog scale:

VAS was used to measure the intensity of pain among the participants. VAS uses points along a line labelled with intensity numbers ranging from 0 to 10, allowing for the measurement of increasing intensity of pain with the number [26]. The severity of pain was measured using GPS. It contains four subscales: activities, feelings, clinical results and individual pain. 20 items are evaluated using an 11-point Likert scale ranging from 0- 10. A sum of 20 items is divided by 2 to achieve the final GPS score between 0-100 [27].

## Pittsburgh sleep quality index:

The PSQI was utilized to assess the overall quality of sleep experienced in the past month. It comprises 19 subject-rated items rated on a 0-3 Likert scale, which are then categorized into 7 components. The total score of all 7 components ranges between 0 to 21, with higher scores indicating poorer sleep quality [28].

## Inflammatory marker:

In the broader population, CRP levels within the range of 0.0 to 5.0 milligrams per liter (mg/L) are generally regarded as normal. Among healthy young adult volunteer blood donors, the median CRP concentration stands at approximately 0.8 mg/L. Notably, the 90th percentile lies at 3.0 mg/L, while the 99th percentile reaches up to 10 mg/L [23, 29]. The normal values for ESR obtained through the Westergren method vary- in healthy individuals under 40 years, it's around 10 mm/h, while for those over 60years, it averages 18 mm/h, though it can extend up to 25 mm/h [30].

## Statistical analysis:

MS-excel was used to collect and screen the outliers and duplications, analysis was done using SPSS version 16 (Statistics Package for Social Sciences). Continuous variables are expressed as median ± SD, while categorical data are expressed in absolute and percentage values (%). The difference in the mean for the status of pain and sleep quality among different severity of FMS individuals based on FIQR was made by the analysis of variance (ANOVA). Association between the variable was assessed using the spearman' coefficient of correlation and p<0.05 was considered as statistically significant.

## Discussion:

The effect of severity of FMS on levels of ESR, CRP, pain and sleep quality was explored in this study. Inflammatory markers, pain intensity and sleep disturbances increased with an increase in FMS severity. CRP levels showed a significantly positive correlation with symptom severity score, PSQI and ESR with CRP levels showing a significantly positive correlation with PSQI components like sleep disturbance and daytime sleepiness (Table 4 and Table 5). However, no associations were found between CRP levels with pain intensity or severity, widespread pain index, or ACR scores. Thus, poor sleep quality and/or sleep disturbances might be associated with elevated CRP levels, as unrefreshed sleep is also considered in the symptom severity score. Pain and sleep have been suggested to have a bidirectional relationship in several studies [31, 32]. Pain causes sleep disruption and disturbed sleep leads to a reduction in pain threshold, increasing spontaneous pain [31]. Sleep deprivation has been demonstrated to increase both spontaneous and evoked pain responses, modulating pain processes [32]. Haack *et al.* have shown that insufficient sleep quantity may lead to an increase in IL-6, exacerbating pain [15]. They showed that inadequate sleep may establish as well as maintain its co-occurrence with pain and inflammation [15]. Meier-Ewert *et al.* Prather *et al.* and Lee *et al.* have shown heightened CRP levels associated with poor sleep patterns like excessive sleep duration, poor sleep quality and sleep deprivation [9, 33, 34].

Our study shows that increasing severity of FMS is associated not only with an increase in pain intensity and severity but also with a reduction in sleep quality and an increase in ESR and CRP levels. Menefee *et al.* have suggested that patients with chronic pain conditions, including fibromyalgia, suffer from greater sleep disturbances compared to general population [3]. However, studies have suggested that FMS is not mainly an inflammatory condition as no inflammatory damage is visible in the joints, muscles, or other tissues [21]. Feinberg *et al.* in a large population-based study, demonstrated that individuals with FMS had a higher level of CRP compared to non-FMS; they explained that the existence of pain comorbidities may contribute to the development of FMS [18]. Though, sleep and mood disturbances attenuated the association of CRP and FMS in their study but could not remove the same [18]. Our study too has explored this association of CRP in FMS individuals but as it was a cross-sectional study design the direction of association of pain, sleep and CRP could not be established. Hodges *et al.* analysed 30,153 UK Biobank participants of chronic and acute musculoskeletal pain and compared it with pain free controls [35]. They observed higher CRP levels in patients with chronic pain compared to those with acute pain or pain-free controls [35]. The sleep scores were poorest in individuals with chronic pain followed by acute pain compared to pain-free individuals, further, there was a negative correlation between CRP levels and sleep scores [35]. In present study too we found a significant association between CRP and PSQI (sleep quality) where higher PSQI scores signify poor sleep quality.

Skarpsno *et al.* examined data of 6356 individuals free of bodily pain, with or without sleeplessness at baseline. Their CRP levels were recorded at baseline and after 8 years of follow-up for the development of widespread/chronic musculoskeletal pain [36]. They suggested interplay of sleeplessness and CRP levels as a risk for any form of widespread/chronic musculoskeletal pain. However, their classification of widespread pain was different from the 1990 ACR diagnostic criteria and also their classification of sleeplessness was based on one self-reported question without specifying sleep latency, actual sleep time *etc.*. In the present study validated sleep quality measure PSQI was used to assess sleep and there was a significant increase in total PSQI score with increasing FMS severity, signifying deterioration of sleep with increasing FMS severity. Studies have suggested a cyclical and self-perpetuating relationship between sleep disturbances and fibromyalgia symptoms. Lack of adequate sleep increases pain sensitivity, while continuous pain further degrades sleep quality [42]. On assessment of sleep components of PSQI with FMS severity, we found a significant increase in scores of sleep quality, sleep latencies, daytime disturbances and sleep disturbances with an increase in FMS severity (Table 3). Bolukbas *et al.* too, reported higher scores for all PSQI components except for sleep medication among their patients with FMS compared to controls [43]. Significant correlations were observed between CRP levels with daytime disturbances and Sleep disturbances components only and not with sleep quality or sleep latency component of PSQI. Irwin *et al.* in their systematic review and meta-analysis on sleep disturbance, sleep duration and inflammation, analyzed 72 studies (n=>50,000) and found that sleep disturbance and longer sleep duration were associated with higher CRP levels [37]. Leng *et al.* found an association of increased CRP with daytime napping in older adults [38]. A study by Mantua *et al.* on young adults found a linear increase in CRP with napping and they suggested interactive effects of excess/insufficient sleep and frequent/infrequent napping with elevated CRP but concluded that these relations depend on both nocturnal and daytime sleep patterns [39]. Thus, a significant positive association of sleep disturbance and daytime disturbance with CRP in present study may be the probable causative culprit for changed pain threshold in chronic pain like FMS (Figure 1 - see PDF).

Gebhardt *et al.* conducted a study involving 72 individuals with acute lumbosciatic pain or chronic low back pain and investigated the relationship between pain intensity and CRP levels, compared to controls [40]. Though no differences were observed at baseline values of CRP in three groups, an initial reduction (at 3 weeks) of CRP was observed in patients with acute lumbosciatic pain, but on further follow-up, no correlation was seen between the pain intensity and /or clinical presentation and CRP levels. Skarpsno *et al.* in another study on patients with chronic low back pain with and without complaints of sleeplessness and insomnia have shown a lessor probability of recovery from chronic pain in those with sleep complaints [41]. They suggested that improvement in sleep may be potentially helpful in improving pain symptomatology. Several studies have shown an association of CRP levels with FMS, but the present study explored the association of FMS severity with sleep quality, levels of CRP and ESR. It shows that CRP levels increase and sleep quality deteriorates with increasing severity of FMS. Further, sleep disturbances have a significant association with an increase in CRP levels. Thus, our findings suggest that disrupted sleep contributes to heightened inflammatory markers in individuals with FMS, which may lead to a change in pain threshold and may play a role in the pathophysiology of FMS (Table 2). Therefore, addressing sleep disturbances may hold promise in mitigating the severity of FMS and reducing inflammation-associated symptoms.

## Funding:

This research received funding from the Department of Science & Technology (DST) - Science and Technology of Yoga and Meditation (SATYAM); New Delhi, India.

## Figures and Tables

**Table 1:** Baseline characters of FMS individuals

**Variables**	**Mean ±SD (N=89)**
Age	38.35±10.04
Gender	F = 87.6%(78)
	M = 12.3%(11)
FIQR	51.097±13.836
CRP	3.38±3.35
ESR	28.23±20.96
WPI	10.66±2.99
SSS (a)	5.26±1.41
Other symptoms (b)	1.75±0.50
Total (a+b)	7.05±1.64
Total ACR	17.73±3.71
VAS	6.91±1.47
GPS	54.76±14.20
PSQI	9.39±3.96
FIQR - Fibromyalgia Impact Questionnaire-Revised;
CRP - C-reactive protein;
ESR - Erythrocyte sedimentation rate;
WPI - Widespread pain Index;
SSS - Severity Scale Score;
Total (a+b)- Severity Scale Score + Other symptoms;
ACR - American College of Rheumatology 2010 criteria;
VAS - Visual Analog Scale;
GPS - Goble pain scale;
PSQI - Pittsburgh Sleep Quality Index.

**Table 2:** Change in pain and sleep with FMS severity

**Variables**	**FIQR grade-1 (N=9)**	**FIQR grade 2 (N=22)**	**FIQR grade 3 (N=44)**	**FIQR grade 4 (N=14)**	**F value**	** P value**
CRP	1.76± 1.59	3.03±2.003	3.63±3.94	4.14±3.35	1.104	0.352
ESR	21.61±18.86	26.18±15.97	28.56±22.42	34.64±24.45	0.804	0.495
WPI	8.22±1.09cd	9.54±2.19c	11.45±3.06ab	11.57±3.15a	5.277	0.002*
SSS(a)	4.11±0.927cd	5.0±1.48	5.45±1.40a	5.85±1.16a	3.647	0.016*
Other symptom (b)	1.66±0.50	1.50±0.51c	1.84±0.47b	1.92±0.47	3.134	0.030*
Total a and b	5.77±0.83cd	6.50±1.53	7.36±1.69a	7.78±1.36a	4.61	0.005*
Total ACR	14±1.11cd	16±2.49cd	18.81±3.69ab	19.35±4.01ab	8.355	0.0001*
FIQR	26±1.87bcd	40.44±4.21acd	55.34±6.17abd	70.61±6.17abc	158.61	0.0001*
VAS	5.44±1.23cd	6.59±1.40	7.15±1.39a	7.57±1.34a	5.359	0.002*
GPS	34.16±10.30bcd	47±14.09bcd	58.94±9.11ab	67.07±9.32ab	23.402	0.0001*
PSQI	5.66±2.23cd	8.09±3.95d	9.97±3.66a	12±3.53ab	6.962	0.0001*
FIQR - Fibromyalgia Impact Questionnaire-Revised;
CRP - C-reactive protein;
ESR - Erythrocyte sedimentation rate;
WPI - Widespread pain Index;
SSS - Severity Scale Score;
Total (a+b)- Severity Scale Score + Other symptoms;
ACR - American College of Rheumatology 2010 criteria;
VAS - Visual Analog Scale;
GPS - Goble pain scale;
PSQI - Pittsburgh Sleep Quality Index.
a - significant difference with FIQR-1;
b - significant difference with FIQR-2;
c - significant difference with FIQR-3;
d - significant difference with FIQR-4.

**Table 3:** Change in the PSQI components with Fibromyalgia severity

**Variables**	**FIQR grade-1 (N=9)**	**FIQR grade 2 (N=22)**	**FIQR grade 3 (N=44)**	**FIQR grade 4 (N=14)**	**F value**	** P value**
Sleep Quality	1.11±0.60d	1.50±0.67	1.72±0.65	2.0±0.78a	3.684	0.015*
Sleep Latency	1.11±0.78cd	2.0±1.06	2.40±0.94a	2.50±0.94a	5.28	0.002*
Sleep Duration	1.11±0.78	1.04±0.95	1.15±0.93	1.14±0.94	0.076	0.973
Sleep efficiency	0.33±0.70	0.31±0.64	0.54±0.81	0.78±1.05	1.116	0.347
Sleep disturbances	1.0±0.70d	0.31±0.71d	1.61±0.65	2.00±0.55ab	5.261	0.002*
Sleep Medication	0.33±0.50	0.72±1.07	0.63±1.10	1.35±1.33	2.032	0.116
Day Time Disturbances	0.66±0.50cd	1.18±0.85c	1.81±0.69ab	1.92±0.82a	9.191	0.0001*
PSQI - Pittsburgh Sleep Quality Index ;
a - significant difference with FIQR-1;
b - significant difference with FIQR-2;
c - significant difference with FIQR-3;
d - significant difference with FIQR-4.

**Table 4:** Correlation of CRP level with other FMS Symptoms

	**Correlation **	**P - value**
FIQR	0.123	0.251
VAS	-0.093	0.384
GPS	0.182	0.089
WPI	0.0001	0.997
SSS (a)	0.279	0.008*
Other Symptom (b)	0.121	0.258
Total (a+b)	0.284	0.007*
Total ACR	0.118	0.272
ESR	0.315	0.003*
PSQI	0.217	0.041*
FIQR - Fibromyalgia Impact Questionnaire-Revised;
CRP - C-reactive protein;
ESR - Erythrocyte sedimentation rate;
WPI - Widespread pain Index;
SSS - Severity Scale Score;
Total (a+b)- Severity Scale Score + Other symptoms;
ACR - American College
of Rheumatology 2010 criteria;
VAS -Visual Analog Scale;
GPS - Goble pain scale;
PSQI - Pittsburgh Sleep Quality Index.

**Table 5:** Correlation of CRP levels with PSQI component domains

**PSQI components**	**Correlation **	**P- value**
Sleep Quality	0.12	0.263
Sleep Latency	0.132	0.218
Sleep Duration	0.068	0.528
Sleep efficiency	-0.055	0.608
Sleep disturbances	0.226	0.033*
Sleep Medication	0.203	0.056
Day Time Disturbances	0.235	0.026*
PSQI - Pittsburgh Sleep Quality Index
